# Draft Genome Sequence of *Pseudomonas aeruginosa* Strain WS394, a Multidrug-Resistant and Highly Cytotoxic Wound Isolate from Chronic Ulcus Cruris

**DOI:** 10.1128/genomeA.01325-14

**Published:** 2014-12-18

**Authors:** Frank-Jörg Vorhölter, Martin Arnold, Daniel Wibberg, Jochen Blom, Anika Winkler, Prisca Viehoever, Andreas Albersmeier, Alexander Goesmann, Sabine Zange, Jürgen Heesemann, Alfred Pühler, Michael Hogardt

**Affiliations:** a CeBiTec (Center for Biotechnology), Universität Bielefeld, Bielefeld, Germany; b Institut für Medizinische Mikrobiologie und Krankenhaushygiene, Universitätsklinikum der Johann-Wolfgang-Goethe-Universität, Frankfurt/Main, Germany; c Max von Pettenkofer-Institut, Ludwig-Maximilians-Universität, München, Germany; d Institut für Mikrobiologie der Bundeswehr, München, Germany

## Abstract

*Pseudomonas aeruginosa* is a frequent human pathogen that increasingly causes chronic infections of nonhealing wounds. Here we present the 6.8 Mb draft genome of strain WS394, a multidrug-resistant chronic ulcer isolate that exhibited outstanding high cell cytotoxicity despite defective secretion of exotoxin U, suggesting a habitat-dependent adaptation process.

## GENOME ANNOUNCEMENT

*Pseudomonas aeruginosa* is a prevalent Gram-negative human pathogen that causes a variety of acute and chronic infections affecting predominantly immunocompromised hosts. Chronic infections such as cystic fibrosis (CF) lung infection due to *P. aeruginosa* are often difficult to treat due to several phenotypic adaptations of this pathogen, including multiple antibiotic resistances (1). Strain WS394 represents a sequential wound isolate from a chronic ulcus cruris of a male patient from Romania. A phenotypic characterization of strain WS394 carried out as described previously (2) revealed the following features: (i) resistance against all available anti-pseudomonal antibiotics except colistin, (ii) polyagglutinating phenotype (with O antisera; Bio-Rad, Munich, Germany), (iii) negative for pyocyanin, (iv) very weak elastase activity, (v) incapability to secrete exotoxin U (ExoU) into culture supernatant, and (vi) high cell cytotoxicity against J774 macrophages. ExoU is a potent cytotoxin with phospholipase A2 activity causing rapid necrotic death in many cell types that may lead to epithelial barrier injury, inhibition of the innate immune response, and impaired wound repair. However, ExoU-secreting, and therefore typically highly virulent, strains were found to be less prevalent in human infections (3).

*P. aeruginosa* genomes range from 5.8 to 7.3 megabases (Mb) in size, with G+C contents of about 66% (http://www.pseudomonas.com). Here, we announce the draft genome of the wound isolate *P. aeruginosa* WS394. To obtain the draft genome sequence, we extracted genomic DNA to construct a paired-end library for shotgun sequencing with the Genome Sequencer FLX (GS FLX) system by means of Titanium technology (Roche), as described recently (4, 5). Standard protocols were followed as per the manufacturer’s instructions. Assembly with the GS *de novo* Assembly software (Newbler) covered 182,064,447 bases from 815,097 aligned individual reads, among them 324,781 paired-end reads. The average size of the paired-end DNA fragments was 2,602 ± 650 bases. The assembly resulted in 27 scaffolds by utilizing the paired-end information. The scaffolds covered 6,754,356 bp, with an average coverage of 26.4× by shotgun reads. The genome had a G+C content of 66.07%.

Automated genome annotation was carried out by means of GenDB software (6). By automated gene prediction, 6,207 protein-coding sequences (CDSs) and 61 RNA-coding genes were identified for *P. aeruginosa* WS394. Comparative analysis employing the EDGAR software (7) confirmed the presence of a well-conserved *exoU* gene and the absence of an *exoS* gene, both of which are substrates of the *Pseudomonas* type III secretion system (T3SS) and are present in a mutually exclusive manner in *P. aeruginosa* genomes (8).

A more detailed analysis of the genome of WS394 will contribute to our understanding on the pathogenicity and habitat-adaptation of chronic wound-infecting pseudomonads and facilitate deeper insights into multidrug resistance of *P. aeruginosa*.

### Nucleotide sequence accession number.

The whole-genome sequencing project for *P. aeruginosa* WS394 has been deposited at EMBL/GenBank/DDBJ under the accession number CBYA000000000. The version described in this paper is the first version.

## References

[1] Hogardt M Heesemann J 2013 Microevolution of *Pseudomonas aeruginosa* to a chronic pathogen of the cystic fibrosis lung. Curr. Top. Microbiol. Immunol. 358:91–118. 10.1007/82_2011_199. 22311171

[2] Hoboth C Hoffmann R Eichner A Henke C Schmoldt S Imhof A Heesemann J Hogardt M 2009 Dynamics of adaptive microevolution of hypermutable *Pseudomonas aeruginosa* during chronic pulmonary infection in patients with cystic fibrosis. J. Infect. Dis. 200:118–130. 10.1086/599360. 19459782

[3] Engel J Balachandran P 2009 Role of *Pseudomonas aeruginosa* type III effectors in disease. Curr. Opin. Microbiol. 12:61–66. 10.1016/j.mib.2008.12.007. 19168385

[4] Schwientek P Szczepanowski R Rückert C Kalinowski J Klein A Selber K Wehmeier UF Stoye J Pühler A 2012 The complete genome sequence of the acarbose producer *Actinoplanes* sp. SE50/110. BMC Genomics 13:112. 10.1186/1471-2164-13-112. 22443545 PMC3364876

[5] Wibberg D Tielen P Blom J Rosin N Schobert M Tüpker R Schatschneider S Spilker D Albersmeier A Goesmann A Vorhölter FJ Pühler A Jahn D 2014 Genome sequence of the acute urethral catheter isolate *Pseudomonas aeruginosa* MH38. Genome Announc. 2(2):e00161-14. 10.1128/genomeA.00161-14. 24625869 PMC3953190

[6] Meyer F Goesmann A McHardy AC Bartels D Bekel T Clausen J Kalinowski J Linke B Rupp O Giegerich R Pühler A 2003 GenDB—an open source genome annotation system for prokaryote genomes. Nucleic Acids Res. 31:2187–2195. 10.1093/nar/gkg312. 12682369 PMC153740

[7] Blom J Albaum SP Doppmeier D Pühler A Vorhölter FJ Zakrzewski M Goesmann A 2009 EDGAR: a software framework for the comparative analysis of prokaryotic genomes. BMC Bioinformatics 10:154. 10.1186/1471-2105-10-154. 19457249 PMC2696450

[8] Yahr TL Wolfgang MC 2006 Transcriptional regulation of the *Pseudomonas aeruginosa* type III secretion system. Mol. Microbiol. 62:631–640. 10.1111/j.1365-2958.2006.05412.x. 16995895

